# Preparing for an influenza season 2021/22 with a likely co-circulation of influenza virus and SARS-CoV-2

**DOI:** 10.2807/1560-7917.ES.2021.26.41.2100975

**Published:** 2021-10-14

**Authors:** Amparo Larrauri, Katarina Prosenc Trilar

**Affiliations:** 1 National Center of Epidemiology, CIBERESP, Carlos III Health Institute, Madrid, Spain; 2 Laboratory for Public Health Virology, National Laboratory for Health, Environment and Food, Ljubljana, Slovenia

Influenza viruses typically circulate in the temperate climates of the northern hemisphere from late fall through early spring and constitute a considerable burden on population health and healthcare systems [1]. The emergence of the severe acute respiratory syndrome corona virus 2 (SARS-CoV-2) in late 2019 and the following coronavirus disease (COVID-19) pandemic had an immense impact on the circulation of influenza viruses and the surveillance of influenza [2]. In spring 2020, countries implemented non-pharmaceutical interventions to mitigate the transmission of SARS-CoV-2 and consecutively observed a rapid decrease in influenza and other respiratory virus infections. A sharp drop in influenza virus-positive specimens occurred in Europe and in the United States (US) in weeks 9 and 10/2020 and the inter-seasonal circulation of influenza in the northern hemisphere was at a historically low level despite higher numbers of tested specimens [3,4]. As we move into the influenza season 2021/22, *Eurosurveillance* has published a Spotlight on influenza 2021 series which highlights a number of relevant aspects that are worth considering ahead of a season in which we may face a co-circulation of SARS-CoV-2 and influenza viruses.

In the *Eurosurveillance* Spotlight on influenza 2021 series, Adlhoch et al. [5] report on epidemiological and virological surveillance of influenza in the 2019/20 season in Europe. An increasing number of countries did not report influenza data from weeks 12 or 13/2020 on, while many countries have maintained their influenza testing activities throughout the COVID-19 pandemic. Data from The European Surveillance System (TESSy) still enabled authors to evaluate the influenza season 2019/20. Influenza A and B viruses were co-circulating, with more influenza A viruses present except in a few countries where influenza B was dominant. There was slightly more circulation of subtype A(H1)pdm09 among influenza A viruses and almost all B viruses were of Victoria lineage. The season peaked in week 5/2020 and soon after, cases started to decline while the number of COVID-19 cases rose sharply and different non-pharmaceutical interventions were introduced to slow down their spread. In addition to these measures, there were changes in healthcare-seeking behaviour and health system organisation. Many sentinel physicians were reallocated for pandemic needs and swabbing of patients with acute respiratory infections (ARI) was reduced or they were sent to COVID-19 diagnostic centres [2].

All this caused a disruption of sentinel surveillance networks at primary care in many countries and contributed to a reduction in influenza-related indicators. Also, some well-established national severe influenza surveillance systems, based on the notification of hospitalised confirmed influenza cases [6], could not be adapted to the emergence of SARS-CoV-2 in a timely manner. Non-sentinel sources also showed a decrease in influenza virus detections despite continued testing, confirming the overall lower influenza circulation since the introduction of COVID-19 related measures. In parallel, many laboratories soon combined detection of SARS-CoV-2 with detection of influenza viruses and integrated the results in their surveillance.

However, despite an unprecedented low influenza activity during 2020 worldwide, it was essential to continue the surveillance of influenza and prepare for the 2020/21 influenza season in the context of the ongoing pandemic during which the importance of well-established disease surveillance networks manifested as crucial. Thus, the World Health Organization (WHO) recommended to adapt influenza surveillance systems and complement them with the COVID-19 surveillance, using severe acute respiratory infection (SARI) and influenza-like illness (ILI)/acute ARI syndromic surveillance systems in primary care, in anticipation of the co-circulation of influenza and SARS-CoV-2 viruses [7]. In Europe, the European Influenza Surveillance Network (EISN) and the Emerging Viral Diseases-Expert Laboratory Network (EVD) were activated immediately to aid in mitigating the pandemic together with the WHO Regional Office for Europe (WHO/Europe) and the Global Influenza Surveillance and Response System (GISRS). The necessary channelling of resources and focus on detecting and limiting infections with SARS-CoV-2 potentially endangers detection and surveillance of influenza and other respiratory viruses. In order to help countries to perform and sustain integrated sentinel surveillance, the European Centre for Disease Prevention and Control (ECDC) and the WHO/Europe published interim guidance on including SARS-CoV-2 testing in surveillance systems in the 2020/21 influenza season [8]. The long-standing experience from influenza surveillance and the surveillance capacities built by countries during the pandemic constitute a solid basis for resilient systems that can respond more effectively and rapidly to public health threats in the future.

Because of the high burden of influenza, it is important for syndromic surveillance to know the contribution of influenza viruses to overall respiratory illness. Belazi et al. [9] estimated the proportion of laboratory-confirmed influenza in Europe among people seeking medical care who were clinically diagnosed with ARI or ILI and were tested for respiratory viruses, including influenza virus. They conducted a meta-analysis of data extracted from studies published between 2004 and 2017 and from sentinel data from TESSy between 2004 and 2018. The proportion of seasonal influenza was 36% in outpatients and 24% in inpatients in the meta-analysis, and 33% vs 24% according to TESSy data. The results show that in Europe, laboratory-confirmed influenza accounts for approximately one third of all ARI for which medical care is sought during the influenza season and where laboratory testing for influenza is undertaken. These findings are of major interest in the context of the ongoing COVID-19 pandemic where changes in operational surveillance aspects, as well as in structures of healthcare systems and/or healthcare-seeking behaviour, may affect the influenza positivity in future seasons. The information provided by Belazi et al. might be an important baseline for comparisons.

Patients with respiratory diseases most often present symptoms that are not sufficiently specific to determine the exact aetiology. Decentralised molecular point-of-care tests (mPOC) have the potential to empower physicians and enable them to make early evidence-based treatment decisions which can improve the prognosis [10]. However, when a diagnostic moves out of the experienced laboratory environment, quality of performance may suffer. For influenza, there is also a fear that data crucially needed for surveillance and public health action will be lost. Benedetti at al [11]. retrospectively studied surveillance data from Denmark over three seasons before and two seasons after mPOC were widely used. They declared instruments using molecular detection and providing test results in less than 3 hours as mPOC. Their findings are promising: the number of tested specimens increased and the share of positive results stayed the same in pre-mPOC and mPOC seasons. Also in mPOC seasons, the share of positive influenza was only slightly lower in mPOC using sites than in central laboratories. That suggests that indication and practice of testing did not change despite availability of mPOC testing. Denmark has a unique national surveillance system that is able to capture results of all performed influenza tests in a single database. Benedetti at al. show how the utilisation of mPOC results in routine surveillance of seasonal influenza can improve the real-time monitoring of influenza virus circulation. With mPOC instruments becoming more affordable, including SARS-CoV-2 in their panels and benefiting from fast results should enable better management of patients in hospitals and other healthcare settings. In countries with less proficient information systems, it is important to establish ways to collect and use the results obtained with mPOC also for surveillance and public health.

The need for integrated testing and surveillance of influenza viruses and SARS-CoV-2 became very clear in the current pandemic. Equally, the detection of other viruses causing respiratory symptoms can indicate unusual situations and provide important information for disease management, like in the case of respiratory syncytial virus (RSV) and the protection of vulnerable infants with monoclonal antibodies. Reagents for molecular detection of multiple respiratory pathogens are available from a number of commercial producers or in the form of in-house-designed multiplex PCR systems as described by Subissi at al [12]. Testing for other respiratory viruses besides influenza shows that they are a frequent cause of severe illness and of respiratory infections outside the usual influenza seasons [13]. Testing 1,791 ILI and 4,774 SARI patients with a multiplex PCR for influenza and other respiratory viruses over four seasons, Subissi at al. found that 17.6% of ILI and 29,9% of SARI patients were positive for one of the other respiratory viruses. Given this large share among SARI patients, it is important to identify the non-influenza virological agent as morbidity and mortality in these patients is high. The burden associated with non-influenza respiratory viruses was especially prominent in children with SARI under 5 years of age. The ability to include non-influenza viruses in influenza surveillance is another benefit of keeping influenza surveillance in good condition. Many laboratories in the influenza surveillance networks have already included detection of SARS-CoV-2 in their repertoire. Understanding the global diversity of respiratory viruses is a crucial element of emerging disease preparedness; non-human CoV and paramyxoviruses have been listed as priority concerns in a WHO initiative on emerging infectious diseases [14].

The trend of a decreased influenza activity seen in the 2019/20 season and in summer 2020 continued in the influenza season 2020/21 when very few influenza viruses were detected in Europe [2] and the US [4]. At the national level, scarce influenza circulation was notified in many countries within the current syndromic sentinel systems. For example, in Spain, the number of specimens tested during the 2020/21 season was higher (n = 4,753) than the average of previous six influenza seasons (n = 4,369). However, only two and one influenza virus were detected in the primary care sentinel ARI surveillance and hospital SARI surveillance systems, respectively, during 2020/21, the first season where these systems were working in Spain (Table).

**Table:** Number of swabs and number and percentage of influenza-positive specimens tested by sentinel systems, before and after the COVID-19 pandemic, Spain, seasons 2014/2015 to 2020/2021

Season	PC - influenza sentinel surveillance system (w40–w20)			PC- ARI surveillance (w40–w38)			Hospital- SARI surveillance (w40–w38)		
	Specimens (n)	Influenza detections (n)	Positivity (%)	Specimens (n)	Influenza detections (n)	Positivity (%)	Specimens (n)	Influenza detections (n)	Positivity (%)
2014/15	5,101	2,779	54.48	NA	NA	NA	NA	NA	NA
2015/16	5,308	2,715	51,15						
2016/17	4,479	2,140	47.78						
2017/18	6,035	3,509	58.14						
2018/19	4,974	2,562	51.51						
2019/20	4,799	2,579	53.74						
2020/21	NA	NA	NA	4,753	2	0.04	6,429	1	0.02

ARI: acute respiratory infection; COVID-19: coronavirus disease; NA: not applicable; n: number; PC: primary care; SARI: severe acute respiratory infection; w: week.

In Slovenia, due to the change in organisation of the primary healthcare system triggered by the pandemic, the number of specimens collected and tested for influenza from the sentinel network at primary care was halved. The average in the previous five seasons was 646 tested specimens and in 2020/21 there were only 324. There were more non-sentinel (mostly hospitals) specimens tested for influenza in the pandemic season 2020/21 (22,616) than the average in the previous five seasons (18,683). There were no influenza viruses detected in sentinel specimens and only one A(H3) in non-sentinel.

In the US, circulation of other respiratory pathogens, including RSV, common human CoV and parainfluenza viruses types 1–4 also decreased in early 2020 and did not increase until spring 2021 [15]. Human metapneumovirus and respiratory adenovirus circulation remained low through May 2021. Rhinovirus and enterovirus detections remained low until May 2020, and then increased to near prepandemic seasonal levels. These different epidemiological patterns of respiratory viruses observed in the US suggest that circulation of respiratory viruses could resume at prepandemic levels after COVID-19 mitigation practices become less stringent.

There is a considerable uncertainty on the influenza virus behaviour in the upcoming 2021/22 season after the historically low levels of circulation since March 2020. It is possible that influenza viruses did not have the opportunity during this period to antigenically evolve. This would put public health in a favourable position because we will not have to face very different virus strains from those that our immune systems know from previous seasons. But it is also possible that due to the lack of exposure to influenza viruses, a proportion of people may have lost their natural immunity which could result in a greater number of influenza infections or even more severe disease [15].

The out-of-season circulation of influenza viruses is a non-negligible possibility as it has occurred recently for RSV in several countries in Europe [16] that experienced an RSV epidemic since mid-May 2021. Some of the contributing factors that were discussed are less stringent public health measures, relaxation of international travel restrictions and viral interference caused by the dominant SARS-CoV-2 that might prevent RSV activity. In this respect, the fact that the 2021/22 influenza season approaches in a critical period of changes in COVID-19 mitigation strategies in many countries represents a cause for concern and highlights the need for strengthening influenza surveillance.

Apart from the public health and social mitigation measures implemented during the COVID-19 pandemic, the high influenza vaccination coverage, mainly among the age groups at greatest risk of COVID-19 and severe influenza, may have also contributed to the low influenza circulation in the 2020/21 season. In Spain, for example, uptake of influenza vaccine increased from an average of 55% in the previous five vaccination campaigns to 64% in the 2020/21 campaign and for healthcare personnel it increased by more than 25% [17].

Given the uncertainties around the intensity of COVID-19 mitigation measures and the return of influenza, it is important to prepare for a possible seasonal influenza virus circulation in the upcoming 2021/22 season. Influenza vaccination is a crucial component of disease prevention that has the potential to prevent significant annual influenza morbidity and mortality [18]. Because of viral antigenic drift, vaccine components change annually in an attempt to adapt them to recent influenza strain circulation. Thus, influenza vaccine effectiveness (VE) studies must be done regularly each season. Among the various study types measuring post-authorisation VE in the population are observational cohort studies, case control studies, pharmaco-epidemiological studies or studies based on computerised medical record systems. Many efforts have been undertaken in the last decade to improve the design of these studies. Baum et al. obtained confounder-adjusted estimates of influenza VE in the elderly population in Finland using a register-based cohort study design based on medical and demographic register data [19]. One of the covariates that they used as confounders is the presence of underlying conditions based on the subject-specific 1-year history of inpatient and outpatient hospital visits before season onset. They also assessed the residual confounding bias using off-season hospitalisation for ARI as a negative control outcome. The authors reported that in eight consecutive seasons, vaccination reduced the hazard of severe influenza disease in those vaccinated by 16% to 48%, but provided no or minimal protection against the unspecific outcome of hospitalisation for ARI, proving that the variables used are sufficient for the control of confounding that may affect influenza VE estimates within register-based cohort studies.

Although influenza activity was historically low during the 2020/21 influenza season, we could experience an influenza activity surge this coming season with relaxed COVID-19 mitigation strategies, increased travel and the reopening of schools and businesses. Influenza vaccination remains the best available preventive measure against influenza particularly in groups recommended vaccination. Even in cases when influenza vaccination does not completely prevent infection, studies have shown [20] it can reduce the duration and severity of illness and help prevent serious complications, including hospitalisation and death. Vaccination is going to be especially important in the 2021/22 season if influenza and SARS-CoV-2 viruses would co-circulate. In this possible scenario, influenza vaccination and the strengthening of an integrated surveillance of influenza viruses and SARS-CoV-2 are critical elements in preparing for a possible resurgence of influenza in the 2021/22 season.

## References

[1] Cassini A Colzani E Pini A Mangen MJ Plass D McDonald SA Impact of infectious diseases on population health using incidence-based disability-adjusted life years (DALYs): results from the Burden of Communicable Diseases in Europe study, European Union and European Economic Area countries, 2009 to 2013. Euro Surveill. 2018;23(16):1700454. 10.2807/1560-7917.ES.2018.23.16.17-00454 29692315 PMC5915974

[2] Adlhoch C Mook P Lamb F Ferland L Melidou A Amato-Gauci AJ Very little influenza in the WHO European Region during the 2020/21 season, weeks 40 2020 to 8 2021. Euro Surveill. 2021;26(11):2100221. 10.2807/1560-7917.ES.2021.26.11.2100221 33739256 PMC7976381

[3] Adlhoch C Pebody R. What to expect for the influenza season 2020/21 with the ongoing COVID-19 pandemic in the World Health Organization European Region. Euro Surveill. 2020;25(42):2001816. 10.2807/1560-7917.ES.2020.25.42.2001816 33094719 PMC7651872

[4] Olsen SJ Azziz-Baumgartner E Budd AP Brammer L Sullivan S Pineda RF Decreased Influenza Activity During the COVID-19 Pandemic - United States, Australia, Chile, and South Africa, 2020. MMWR Morb Mortal Wkly Rep. 2020;69(37):1305-9. 10.15585/mmwr.mm6937a6 32941415 PMC7498167

[5] Adlhoch C Sneiderman M Martinuka O Melidou A Bundle N Fielding J Spotlight influenza: The 2019/20 influenza season and the impact of COVID-19 on influenza surveillance in the WHO European Region. Euro Surveill. 2021;26(40):2100077. 10.2807/1560-7917.ES.2021.26.40.2100077 34622760 PMC8511754

[6] Spanish Influenza Surveillance System. [Annual influenza report in Spain, season 2019-20.] Madrid: Instituto de Salud Carlos III; 2020. Spanish. Available from: https://vgripe.isciii.es/documentos/20192020/InformesAnuales/Informe_Vigilancia_GRIPE_2019-2020_03092020.pdf

[7] World Health Organization (WHO). Maintaining surveillance of influenza and monitoring SARS-CoV-2 – adapting Global Influenza surveillance and Response System (GISRS) and sentinel systems during the COVID-19 pandemic: Interim guidance. Geneva: WHO; 2020. Available from: https://www.who.int/publications/i/item/maintaining-surveillance-of-influenza-and-monitoring-sars-cov-2-adapting-global-influenza-surveillance-and-response-system-(gisrs)-and-sentinel-systems-during-the-covid-19-pandemic

[8] European Centre for Disease Prevention and Control (ECDC), WHO Regional Office for Europe. (WHO/Europe). Operational considerations for influenza surveillance in the WHO European Region during COVID-19: interim guidance. Stockholm, Geneva: ECDC, WHO/Europe; 2020. Available from: https://www.ecdc.europa.eu/en/publications-data/operational-considerations-influenza-surveillance-european-region-during-covid-19

[9] Belazi S Olsen SJ Brown C Green HK Mook P Nguyen-Van-Tam J Spotlight influenza: Laboratory-confirmed seasonal influenza in people with acute respiratory illness: a literature review and meta-analysis, WHO European Region, 2004 to 2017. Euro Surveill. 2021;26(39):2000343. 10.2807/1560-7917.ES.2021.26.39.2000343 34596019 PMC8485580

[10] Kim H Huh HJ Park E Chung DR Kang M. Multiplex Molecular Point-of-Care Test for Syndromic Infectious Diseases. Biochip J. 2021;15(1):14-22. 10.1007/s13206-021-00004-5 33613852 PMC7883532

[11] Benedetti G Krause TG Schneider UV Lisby JG Voldstedlund M Bang D Spotlight influenza: Influenza surveillance before and after the introduction of point-of-care testing in Denmark, season 2014/15 to 2018/19. Euro Surveill. 2021;26(37):2000724. 10.2807/1560-7917.ES.2021.26.37.2000724 34533117 PMC8447826

[12] Subissi L Bossuyt N Reynders M Gérard M Dauby N Lacor P Spotlight influenza: Extending influenza surveillance to detect non-influenza respiratory viruses of public health relevance: analysis of surveillance data, Belgium, 2015 to 2019. Euro Surveill. 2021;26(38):2001104. 10.2807/1560-7917.ES.2021.26.38.2001104 34558405 PMC8462033

[13] Tai C-C, Tsai C-H, Huang Y-H, Lee C-L, Chen H-P, Chan Y-J. Detection of respiratory viruses in adults with respiratory tract infection using a multiplex PCR assay at a tertiary center. J Microbiol Immunol Infect. 2020;S1684-1182(20)30176-6. 10.1016/j.jmii.2020.07.020 32826192 PMC7422795

[14] World Health Organisation (WHO). 2018 annual review of diseases prioritized under the Research and Development Blueprint. Geneva: WHO; 2018. Available from: https://www.who.int/docs/default-source/blue-print/2018-annual-review-of-diseases-prioritized-under-the-research-and-development-blueprint.pdf?sfvrsn=4c22e36_2

[15] Olsen SJ Winn AK Budd AP Prill MM Steel J Midgley CM Changes in Influenza and Other Respiratory Virus Activity During the COVID-19 Pandemic - United States, 2020-2021. MMWR Morb Mortal Wkly Rep. 2021;70(29):1013-9. 10.15585/mmwr.mm7029a1 34292924 PMC8297694

[16] van Summeren J Meijer A Aspelund G Casalegno JS Erna G Hoang U Low levels of respiratory syncytial virus activity in Europe during the 2020/21 season: what can we expect in the coming summer and autumn/winter? Euro Surveill. 2021;26(29):2100639. 10.2807/1560-7917.ES.2021.26.29.2100639 34296672 PMC8299745

[17] Ministry of Health. [Results of influenza vaccination coverage, 2020-2021 season.] Madrid: Ministry of Health; 2021. Spanish. Available from: https://www.mscbs.gob.es/profesionales/saludPublica/prevPromocion/vacunaciones/calendario-y-coberturas/coberturas/home.htm

[18] World Health Organization (WHO). Vaccines against influenza WHO position paper – November 2012. Wkly Epidemiol Rec. 2012;87(47):461-76. 23210147

[19] Baum U Kulathinal S Auranen K. Spotlight influenza: Estimation of influenza vaccine effectiveness in elderly people with assessment of residual confounding by negative control outcomes, Finland, 2012/13 to 2019/20. Euro Surveill. 2021;26(36):2100054. 10.2807/1560-7917.ES.2021.26.36.2100054 34505568 PMC8431990

[20] Casado I Domínguez A Toledo D Chamorro J Force L Soldevila N Effect of influenza vaccination on the prognosis of hospitalized influenza patients. Expert Rev Vaccines. 2016;15(3):425-32. 10.1586/14760584.2016.1134328 26690376

